# Cyclic Distraction–Compression Dynamization Technique Enhances the Bone Formation During Distraction Osteogenesis

**DOI:** 10.3389/fbioe.2021.810723

**Published:** 2022-01-18

**Authors:** Yanshi Liu, Feiyu Cai, Kai Liu, Jialin Liu, Xiaoxu Zhang, Aihemaitijiang Yusufu

**Affiliations:** ^1^ Department of Trauma and Microreconstructive Surgery, The First Affiliated Hospital of Xinjiang Medical University, Urumqi, China; ^2^ Department of Prosthodontics, The First Affiliated Hospital of Xinjiang Medical University, Urumqi, China; ^3^ School of Public Health, Xinjiang Medical University, Urumqi, China

**Keywords:** amplitude, bone formation, cyclic distraction–compression, distraction osteogenesis, rate, accordion maneuver

## Abstract

**Background:** Interfragmentary movements have benefits in the improvement of bone formation during distraction osteogenesis (DO). Although several clinical studies reported positive outcomes regarding the application of the cyclic distraction–compression (CDC) dynamization technique in cases with poor bone formation during DO, they are mostly anecdotal without a detailed description. The purpose of this study was to investigate the effectiveness and potential mechanism of different amplitudes and rates of the CDC technique on bone regeneration in a rat femur DO model.

**Methods:** A total of 60 adult male Sprague-Dawley rats underwent right femoral mid-diaphysis transverse osteotomy and were randomly and evenly divided into Control (no manipulation), Group1 (CDC therapy), Group2 (CDC therapy with larger amplitude), and Group3 (CDC therapy with a slower rate) after distraction. The CDC technique was performed during the middle phase of the consolidation period according to different protocols. Animals were sacrificed after 4 and 6 weeks of consolidation. The process of bone formation was monitored by digital radiographs, and the regenerate bone was evaluated by micro-computed tomography (micro-CT), biomechanical test, and histological analysis. The serum contents of hypoxia-inducible factor (HIF)-1α and vascular endothelial growth factor (VEGF) were measured by enzyme-linked immunosorbent assay (ELISA).

**Results:** Bone regeneration after the CDC technique was improved significantly during DO. The digital radiograph, micro-CT, histomorphological analysis, and biomechanical evaluation showed better effects regarding volume, continuity, and mechanical properties of the regenerate bone in Group2 and Group3 when compared to Group1. The angiogenic and osteogenic markers were more highly expressed in Group2 and Group3 than in Group1 according to the immunohistochemical analysis. As for ELISA, the serum contents of HIF-1α and VEGF were also increased after the CDC technique, especially in Group2 and Group3.

**Conclusion:** The CDC dynamization technique has benefits on the improvement of bone formation during DO, and the mechanism may be due to tissue hypoxia activating the HIF pathway followed by the augmentation of osteogenic–angiogenic coupling. Better outcomes may be achieved by moderately increasing the amplitude and slowing down the rate of the CDC technique.

## Introduction

Distraction osteogenesis (DO), firstly described by Ilizarov (1989a, 1989b, 1990) in the 1950s, has become a widely applied technique in orthopedic and reconstructive surgeries for limb lengthening, deformity correction, and bone defect caused by trauma, infection, or malignancy (Biz et al., 2021; Dahl and Morrison, 2021; El-Alfy et al., 2021). DO is a bone-regenerative process in which gradual distraction generates two vascularized bone surfaces, and the new bone tissue is generated in the gap between the two progressively distracted bony segments (Ilizarov, 1989a; Ilizarov, 1989b). The principle is derived from the intrinsic capability of the bone to regenerate in a controlled mechanical environment, including advantages of avoidance of local or distant donor site morbidity, and concurrent generation of tissues using local endogenous substrates (Ai-Aql et al., 2008). Additionally, DO is considered the best type of *in vivo* bone tissue engineering technique (Makhdom and Hamdy, 2013).

Although satisfactory outcomes have been achieved in most cases, the most important limitation of DO is the absence or delayed callus formation in the distraction gap, which can prolong the duration with a bulky frame needed for the regenerate bone to finally consolidate, resulting in additionally unfavorable psychological impact and negative complications (Paley, 1990; Spiegl et al., 2013; Biz et al., 2021). Numerous previous studies have been described to ensure successful regenerate formation during DO, such as systemic or local addition of several pharmacological agents, osteogenic factors, or bone formation-inducing proteins (Wang et al., 2021a; Wang et al., 2021b; Pan et al., 2021). However, most factors are difficultly applied in clinical practice due to regulatory controls, uncertain therapeutic efficacy, and high cost (Alzahrani et al., 2018).

As generally accepted, the mechanical environment plays a vital role in bone formation (osteogenesis and chondrogenesis) and that bones adapt to the mechanical loads they carried in terms of modeling, remodeling, and regeneration (Huang and Ogawa, 2010). Efforts have been made to enhance bone formation via physical stimulation during DO, finding that applying compression to the distraction gap has positive effects (Mofid et al., 2002; Pacicca et al., 2002; Ozgul et al., 2012; Xu et al., 2018). Compared with other alternative management such as pharmacologic treatment, physical therapy is easier, safer, and more practical in the clinic. Therefore, it is currently considered that mechanical stimulation is an important factor for the consolidation and maturation of callus in DO (Pacicca et al., 2002; Kwon et al., 2020).

Modalities of compressive forces for enhancing bone regeneration in DO include increasing weight bearing on the suffered limb, fixator dynamization, overdistraction and then shortening, and cyclic distraction–compression (CDC) (Mofid et al., 2002; Robling et al., 2002; Mori et al., 2006; Claes et al., 2008). The CDC dynamization technique, which is named as an accordion maneuver, is originally described by Ilizarov (1990) to accelerate bone formation in DO. Although lots of clinical success of CDC in the treatment of poor regenerate have been reported, they are mostly anecdotal with a poor description, and the optimal pattern of CDC stays uncertain (Inan et al., 2005; Madhusudhan et al., 2008; Makhdom et al., 2015; Zhang et al., 2017; Liu et al., 2020; Meselhy et al., 2021). Several experimental studies performed on animals have provided some limited details. Mofid et al. (2002) reported that daily CDC for 3 weeks during the consolidation period has benefits for bone formation in the rabbit model. Claes et al. (2008) observed higher bone formation after temporary distraction and compression in fracture healing. Xu et al. (2018) confirmed that the accordion maneuver during the middle phase of the consolidation period effectively accelerated new bone formation. Even both clinical and experimental published data have demonstrated the positive benefits of CDC in the enhancement of bone regeneration, but there are little data available regarding the effects of different amplitudes and rates of CDC on bone formation.

These practical constraints ground the premise of the present study. The purpose of this study was to investigate the effectiveness and potential mechanism of different amplitudes and rates of CDC dynamization technique on bone regeneration in a rat femur DO model.

## Materials and Methods

### Animals

Adult male Sprague-Dawley rats weighing approximately 420 g were provided by the Experimental Animal Centre of Xinjiang Medical University [license no. SYXK (Xin) 2018-0003]. Rats were maintained in a pathogen-free environment and housed in a light/dark and temperature-controlled room. During a 7-day acclimation period prior to surgery, rats had free access to standard laboratory chow and sterile water. All procedures that involved animals were performed in accordance with the Guide for the Care and Use of Laboratory Animals of the First Affiliated Hospital of Xinjiang Medical University. Ethics approval was obtained from the Animal Ethics Committee of the First Affiliated Hospital of Xinjiang Medical University (no. IACUC-20200318-82).

### Surgical Procedures and Postoperative Care

**FIGURE 1:**
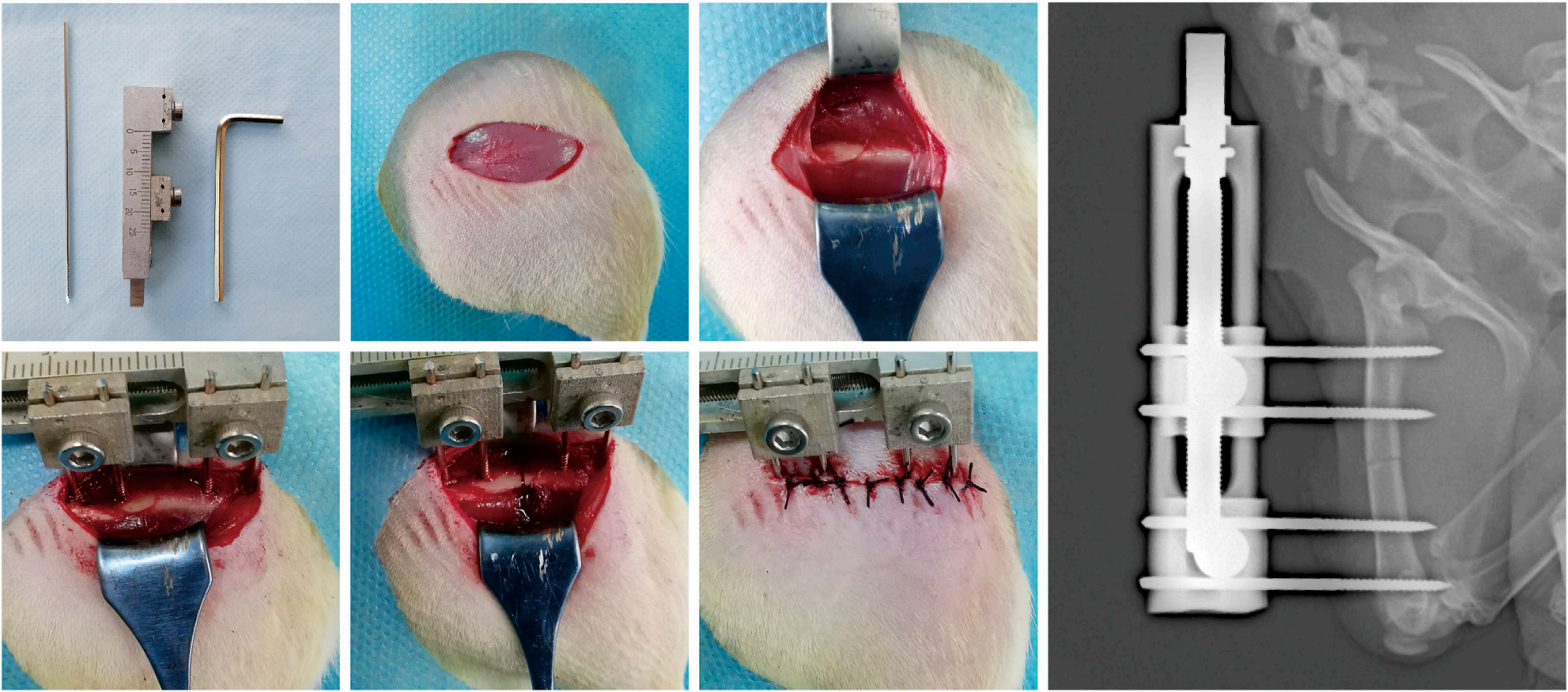
The surgical procedures for the rat right femur model of DO.

All surgical operations were performed by the same skilled surgical team. A total of 60 rats were used in the present study. The rats were anesthetized with 2% pentobarbital sodium (3 mg/100 g). A preoperative dose of benzyl penicillin was administered for infection prophylaxis. A custom monolateral distraction external fixator (designed and manufactured by the School of Mechanical Engineering, Xinjiang University) was installed on the right femur using four stainless-steel self-tapping screws under sterile conditions followed by a mid-diaphysis transverse osteotomy (Figure 1).

Daily pin site care was performed using the antibiotic solution. Intramuscular injection of benzyl penicillin was conducted daily during the postoperative 3 days in all experimental rats for infection prevention. Rats were housed one per cage and free to move, and with free access to chow and water.

### CDC Protocol

**FIGURE 2:**
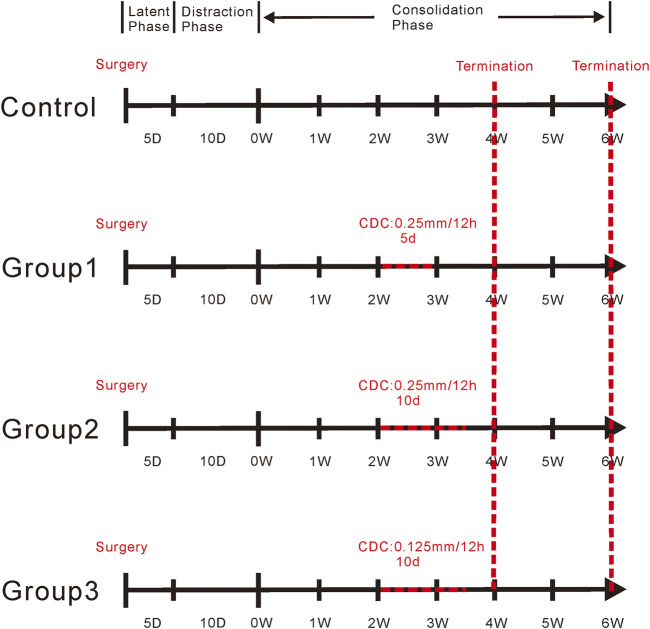
The schematic diagram of the CDC protocol. The CDC technique was performed in the middle phase of bone consolidation (started at week 3 in the consolidation period). Control, no any interventions; Group1, 2.5-day compression and 2.5-day distraction at a rate of 0.25 mm/12 h for 5 days; Group2, 5-day compression and 5-day distraction at a rate of 0.25 mm/12 h for 10 days; Group3, 5-day compression and 5-day distraction at a rate of 0.125 mm/12 h for 10 days.

After a 5-day latency, the distraction was started at a rate of 0.25 mm/12 h for 10 days, producing a cumulative final gap distance of 5.0 mm followed by a consolidation duration of 6 weeks. Rats were randomly divided into four groups according to the pattern of the CDC technique. The CDC technique was performed in the middle phase of bone consolidation (started at week 3 in the consolidation period) based on a previous study (Xu et al., 2018). The details of the CDC protocol were as follows: Control (*n* = 15), no interventions; Group1 (*n* = 15), 2.5-day compression and 2.5-day distraction at a rate of 0.25 mm/12 h for 5 days; Group2 (*n* = 15), 5-day compression and 5-day distraction at a rate of 0.25 mm/12 h for 10 days; and Group3 (*n* = 15), 5-day compression and 5-day distraction at a rate of 0.125 mm/12 h for 10 days. Such parameters were defined for investigating the effect of amplitude (compared with that in Group1, the amplitude was increased in Group2) and rate (compared with that in Group 1, the rate was slowed down in Group3) of CDC on bone formation (Figure 2).

Rats were sacrificed after 4 and 6 weeks of consolidation (*n* = 6 per group at 4 weeks and *n* = 9 per group at 6 weeks). Bilateral femora without soft tissues were harvested for further analysis.

### Digital Radiographic Evaluation

After a brief anesthesia by isoflurane, the same digital X-ray apparatus (HF400VA, MIKASA X-RAY Co., Ltd., Tokyo, Japan) and conditions (44 kV, 4.5 mA) were used for weekly anteroposterior (AP) X-ray examination of the distraction zone until sacrifice of all experimental rats.

### Micro-computed Tomography (Micro-CT)

When the 6-week consolidation was terminated, micro-CT imaging (Al + Cu filter, voxel size 0.9 μm, 80 kV, 313 μA for 0.203 s; SkyScan 1176, Bruker, Rheinstetten, Germany) was performed to quantitatively evaluate the microstructure of the distraction zone (*n* = 3 per group). The scanned images were optimized and reconstructed by SkyScan NRecon software, and the three-dimensional (3D) algorithms in SkyScan CTAn software were performed to analyze these images according to the manufacturer’s instructions. The distraction region, which was surrounded by the outlined periosteum from the proximal and distal ends, was defined as the region of interest (ROI) (Perrien et al., 2012). Only the bone within the ROI was selected for the measurement of bone mineral density (BMD) and bone volume/total tissue volume (BV/TV).

### Biomechanical Test

After 6 weeks of consolidation, a three-point bending test was performed within 24 h at room temperature to evaluate the mechanical properties of samples (*n* = 3 per group) using a three-point bending apparatus (RGM-3005T, ShenZhen Reger Instrument Co., Ltd., China). Before the test, all external fixators, screws, and surrounding soft tissues were removed, and the unoperated femurs were used as controls. The femur long axis was aligned perpendicular to the blades with the span set as 18 mm, and the distraction zone of specimens was constantly loaded in the AP direction at a loading rate of 0.5 mm/min until failure. Ultimate load, modulus of elasticity (E-modulus), energy to failure, and stiffness were recorded and calculated by the built-in software (REGER, ShenZhen Reger Instrument Co., Ltd., China) and normalized to the contralateral femur.

### Histomorphological and Immunohistochemical Analysis

All samples were fixed in 10% formalin buffer for 48 h and then transferred to 75% ethanol for further analysis. After termination at each time point, three specimens from each group were randomly selected for gradient alcohol dehydration and xylene defatting and then embedded in methyl methacrylate. Sections that were 10 μm thick were cut with a hard-tissue microtome (HistoCore AUTOCUT, Leica, Wetzlar, Germany). The sections underwent Von Kossa, Masson Trichrome, Goldner Trichrome, and Safranin O staining for histomorphometric observation.

After radiological evaluation, the other three specimens per group at each time point were decalcified in 10% ethylenediaminetetraacetic acid solution for 4 weeks, and then sample dehydration, transparency, and paraffin embedding were successively performed. Sections (5 μm) were cut using a microtome (RM2135, Leica, Wetzlar, Germany) for immunohistochemistry staining. After deparaffinization in xylene and rehydration in a graded series of alcohol, immunohistochemistry staining was performed following a standard protocol. Hydrogen peroxide (0.3%) was used to quench the endogenous peroxidase activity for 20 min. Antigen retrieval was performed in 0.4% pepsin solution at 37°C for 25 min followed by blocking with 5% goat serum at 37°C for 30 min. Subsequently, sections were incubated with primary antibodies anti-hypoxia-inducible factor (HIF)-1α (1:100, ab216842, Abcam, Cambridge, United Kingdom), anti-vascular endothelial growth factor (VEGF) (1:100, sc7269, Santa Cruz, CA, United States), anti-runt-related transcription factor 2 (RUNX2) (1:100, sc390351, Santa Cruz, CA, United States), anti-osterix (Osx) (1:400, ab209484, Abcam, Cambridge, United Kingdom), anti-osteocalcin (OCN) (1:100, 23418-1-AP, ProteinTech, Wuhan, China), and anti-osteopontin (OPN) (1:100, 22952-1-AP, ProteinTech, Wuhan, China) overnight at 4°C. After incubation in secondary antibody (PV6000, ZSGB-BIO, Beijing, China) at 37°C for 1 h, a horseradish peroxidase–streptavidin system (ZLI-9019, ZSGB-BIO, Beijing, China) was used for signal detection, followed by counterstaining with hematoxylin. Under a magnification of ×200, three fields in the regenerate zone of each section were randomly selected for analysis (DP26, OLYMPUS, Japan). The positively stained area or cells were semi-quantitatively analyzed by Image Pro Plus 6.0 software.

### Blood Collection and Enzyme-Linked Immunosorbent Assay (ELISA) Analysis

Blood samples (2 ml) were collected by cardiac puncture immediately after termination at each time point (*n* = 6 per group). Serum was obtained via centrifugation at 1,800 rpm for 15 min at room temperature. ELISA kits were used to evaluate the serum content of HIF-1α (JL20959, Shanghai Jianglai Industrial Limited by Share Ltd., Shang Hai, China) and VEGF (JL21369, Shanghai Jianglai Industrial Limited by Share Ltd., Shang Hai, China) according to the manufacturer’s instructions.

### Statistical Analysis

Statistical analysis was performed by SPSS 22.0 (SPSS Inc., Chicago, IL, United States). All continuous variables were expressed as mean ± standard deviation (SD). Normality of the data was evaluated by the Shapiro–Wilk test. The statistical differences were assessed by the independent-samples *t*-test or Mann–Whitney *U*-test between two specific groups. A statistically significant difference was set at *p* < 0.05. Graphs were derived from GraphPad Prism v.6.0 (GraphPad Inc., San Diego, CA, United States).

## Results

### Radiographic Evaluation

During the experiments, rats completely recovered from surgical procedures and survived up to the termination of the experiment. All rats achieved normal ambulation, and there was no significant difficulty in daily activity. Additionally, during the procedures of the CDC technique, there were no significant negative effects on the surrounding soft tissues due to the small CDC magnitude difference of only 1.25 mm among the experimental groups (Group1–Group3).

**FIGURE 3:**
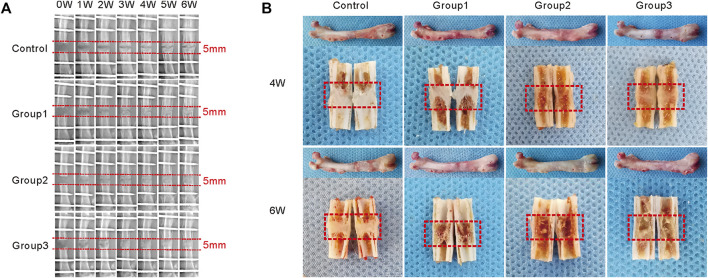
Different patterns of the CDC technique affect bone regeneration during DO. **(A)** X-ray images of the distraction regenerate weekly until a 6-week consolidation duration is terminated. **(B)** The general image of specimens after 2, 4, and 6 weeks of consolidation.

**FIGURE 4:**
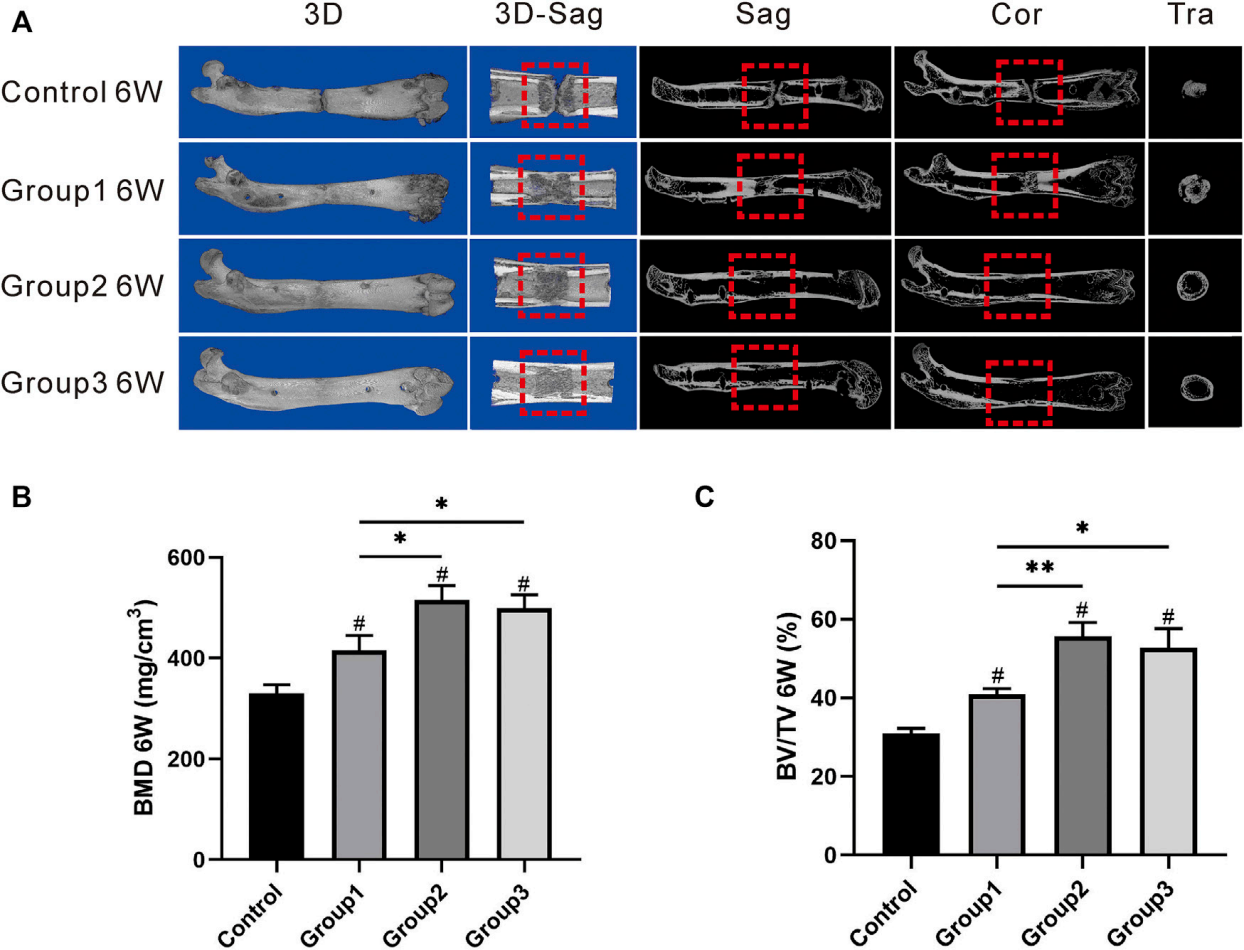
Results of micro-CT evaluation demonstrating promotion in regenerate quality after the CDC technique. **(A)** Representative 3D micro-CT images of the distraction zone at the termination of the 6-week consolidation. **(B**, **C)** Quantitative evaluation of BMD and BV/TV, manifesting that the two values in Group2 and Group3 were significantly higher than those in Group1 (^#^ *p* < 0.05 vs. Control, *p < 0.05 vs. Group1, **p < 0.01 vs. Group1).

Consolidation progression of the distraction regenerate was monitored weekly by digital radiographs. As shown in the representative images, there were no significant differences in bone formation in the first 2 weeks of the consolidation phase between the four groups (Figure 3A). After the CDC technique at week 3, bone regeneration was greater in Group1, Group2, and Group3. When the CDC was completed at week 4, a significant enhancement of regenerate consolidation was observed in Group2 and Group3. At the termination of the 6-week consolidation, there was a remaining gap between the proximal and distal bony ends in Control, while the bone union was achieved in Group1, Group2, and Group3. In addition, compared with that in Group1, the newly regenerated bone was greater in Group 2 and Group3 in terms of the volume and continuity of the callus. Similar results were observed in the general inspection of the dissected specimens (Figure 3B) as well as the micro-CT examination after the 6-week consolidation (Figure 4A); primary recanalization of the medullary cavity was achieved in Group2 and Group3.

### Micro-CT Evaluation

After 6 weeks of consolidation, the representative images of micro-CT manifested the continuity of the marrow cavity in Group2 and Group3, which was almost completely remodeled; bone union without recanalization of the medullary cavity was achieved in Group1; and a narrow gap in the distraction zone was observed in Control (Figure 4A). Additionally, there was statistical significance in terms of BMD value in Group1 (415.97 ± 29.27 mg/cm^3^), Group2 (515.87 ± 28.16 mg/cm^3^), and Group3 (499.77 ± 25.74 mg/cm^3^) when compared with Control (330.23 ± 17.26 mg/cm^3^) (*p* < 0.05), and the BMD value was higher in Group2 and Group3 than in Group1 (*p* < 0.05) (Figure 4B). As for the value of BV/TV, the significant differences were also shown in Group1 (40.87 ± 1.50%), Group2 (55.73 ± 3.45%), and Group3 (52.77 ± 4.84%) when compared with Control (30.87 ± 1.35%) (*p* < 0.05); the BV/TV value in Group2 and Group3 was also obviously higher than that in Group1 (*p* < 0.05) (Figure 4C). The results revealed that the CDC technique has benefits in the promotion of bone regeneration during DO; better outcomes may be achieved by increasing the amplitude and slowing down the rate of the CDC technique.

### Mechanical Property Analysis

**FIGURE 5:**
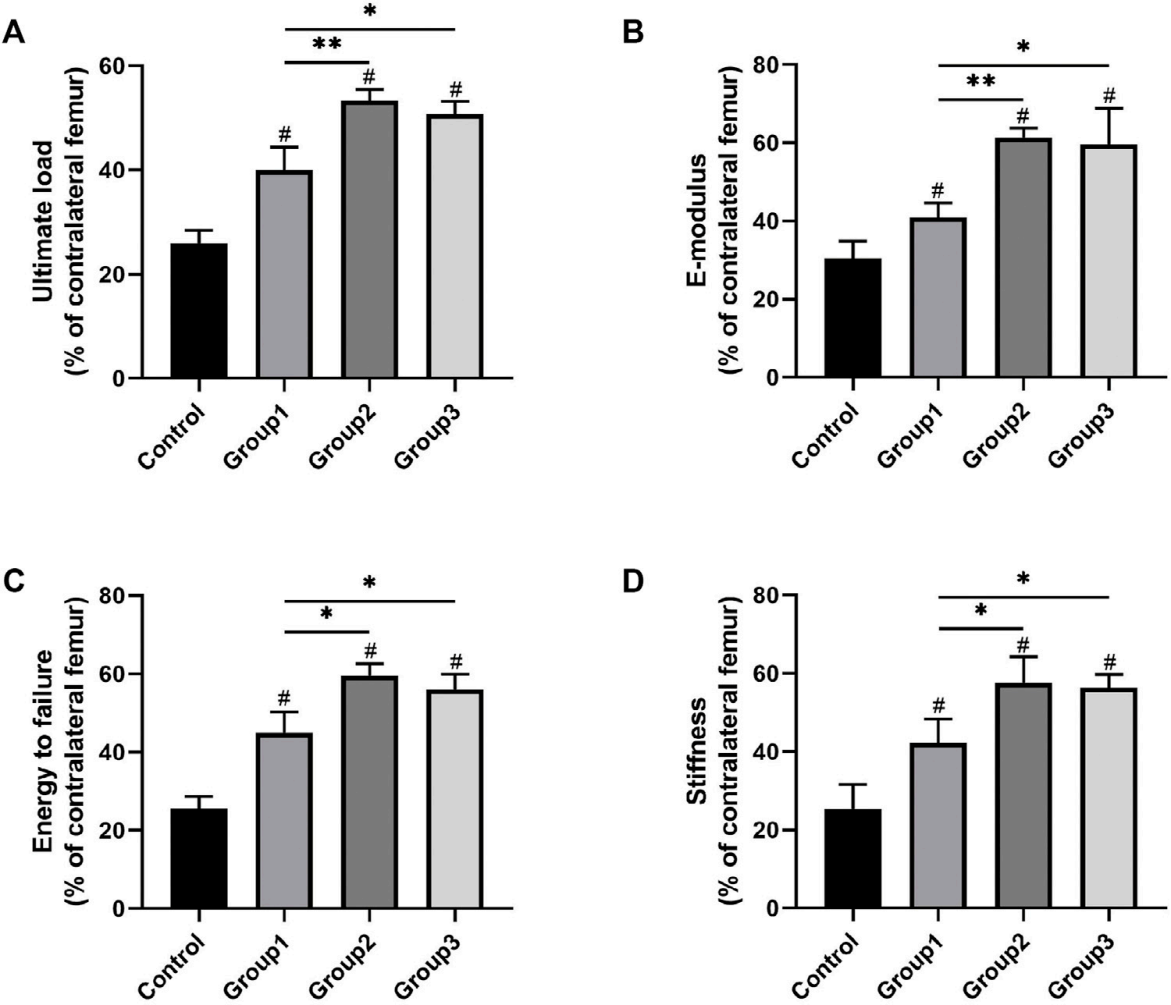
Results of mechanical properties and values were normalized to the contralateral femur (^#^ *p* < 0.05 vs. Control, *p < 0.05 vs. Group1, **p < 0.01 vs. Group1).

The mechanical properties of samples after 6 weeks of consolidation were evaluated by the three-point bending test. There were better outcomes after the CDC technique was applied in Group1, Group2, and Group3 compared with Control (*p* < 0.05). In addition, the results in Group2 and Group3 revealed obvious improvement regarding ultimate load (53.35 ± 2.15% and 50.73 ± 2.43%, respectively), E-modulus (61.40 ± 2.34% and 59.68 ± 9.22%, respectively), energy to failure (59.51 ± 3.13% and 56.04 ± 3.84%, respectively), and stiffness (57.54 ± 6.66% and 56.33 ± 3.37%, respectively) when compared to Group1 (40.02 ± 4.42%, 40.93 ± 3.67%, 44.98 ± 5.29%, and 42.31 ± 5.96%, respectively) (*p* < 0.05) (Figure 5).

### Histological Assessment

**FIGURE 6:**
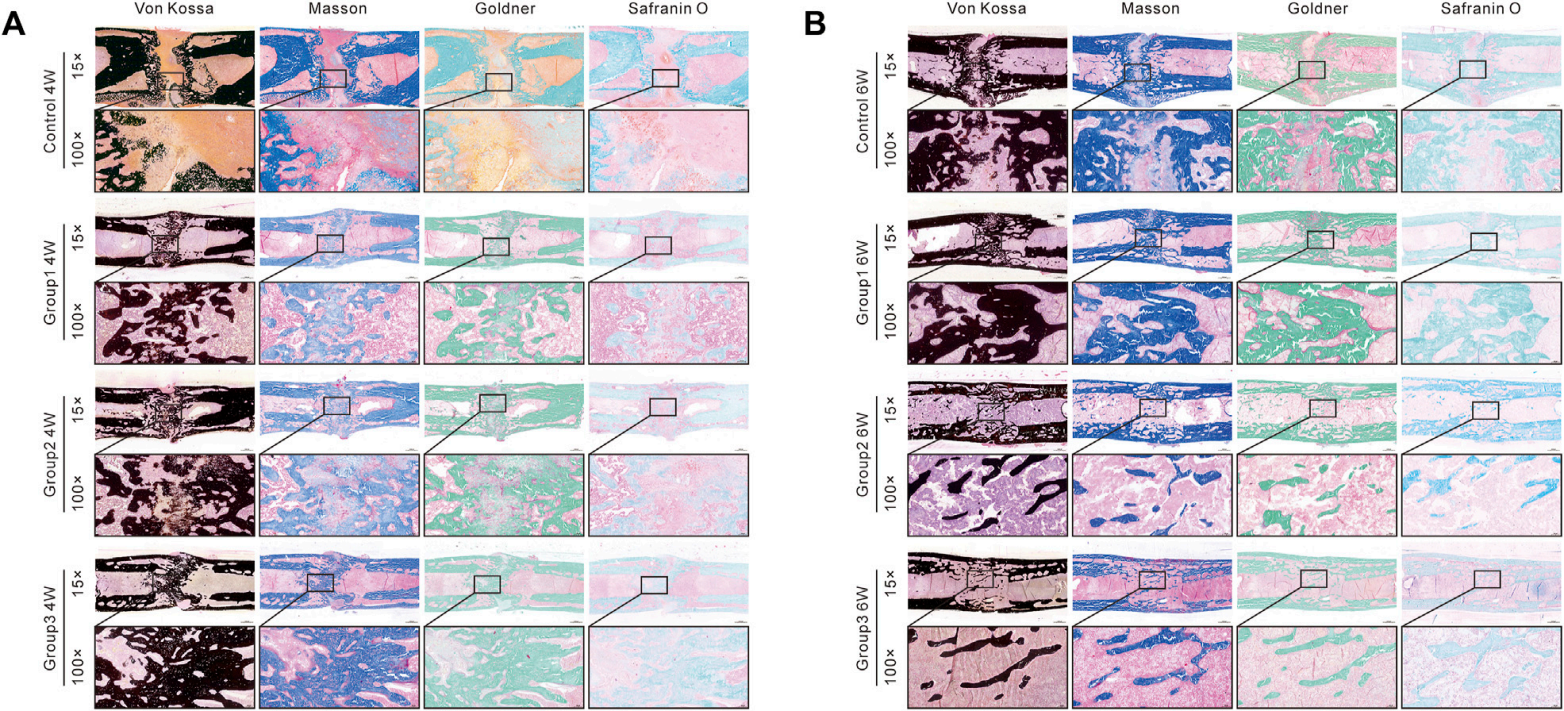
Histomorphological analysis of bone regeneration during the consolidation period. Von Kossa, Masson Trichrome, Goldner Trichrome, and Safranin O staining indicated the enhanced bone regeneration in Group1, Group2, and Group3 (especially in Group2 and Group3).

The histomorphological characteristics of the undecalcified samples were assessed by Von Kossa, Masson Trichrome, Goldner Trichrome, and Safranin O staining. Von Kossa staining manifested that there remained an evident gap in the distraction zone in Control after 4 weeks of consolidation, while better quality and volume of newly regenerated callus were observed in the other three groups, especially in Group2 and Group3 (Figure 6A). At the termination of the 6-week consolidation, a narrow gap still existed in the distraction zone in Control, while the bone union was achieved in Group1, Group2, and Group3. Additionally, complete remodeling with recanalization of the medullary cavity was achieved in Group2 and Group3. Similar results were observed in Masson Trichrome, Goldner Trichrome, and Safranin O staining, demonstrating that the bone formation was accelerated significantly in Group2 and Group3. Moreover, the chondrocytes (cartilage) were evidently observed at the center of the distraction zone in Control after 6 weeks of consolidation based on Safranin O staining, indicating that there remained incompletely mineralized regenerated bone (Figure 6B).

**FIGURE 7:**
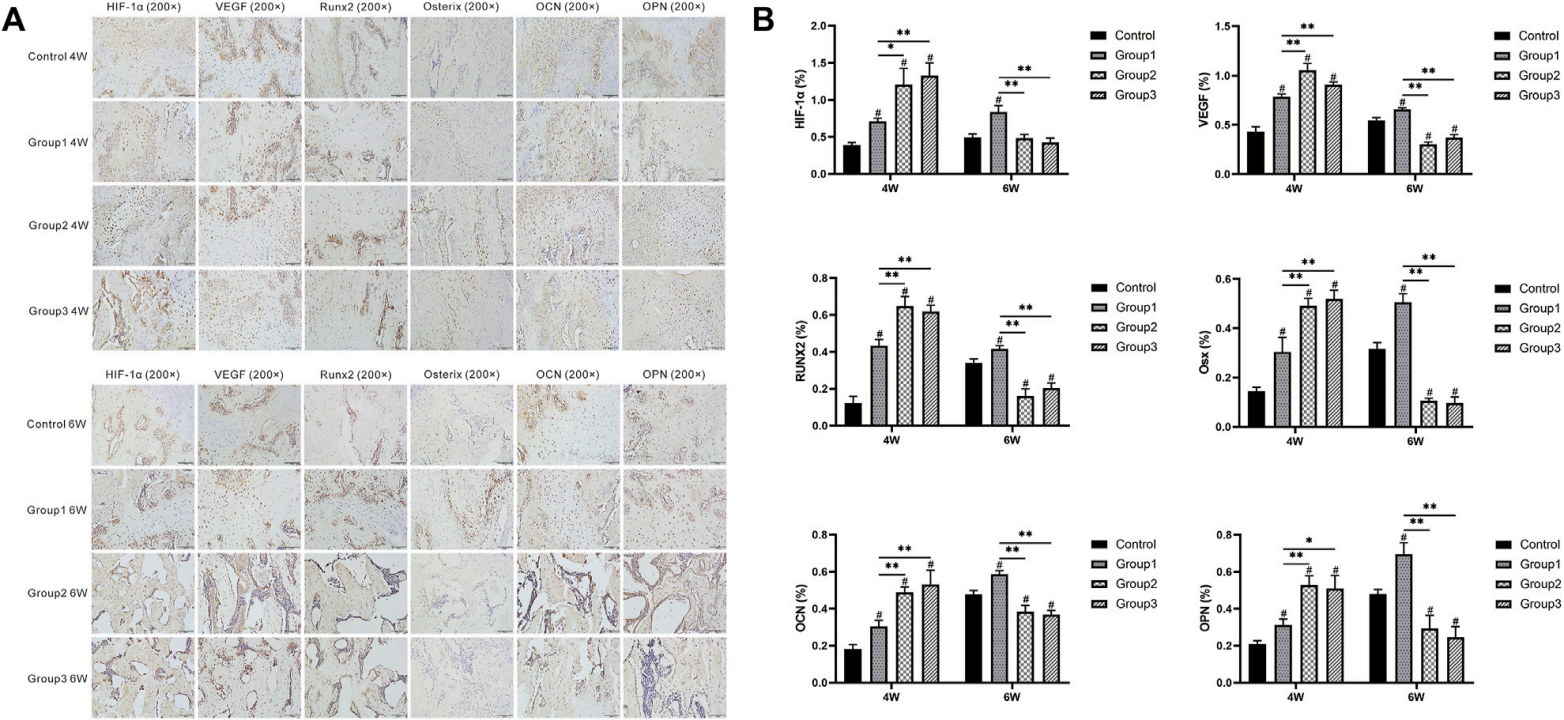
Immunohistochemistry images of HIF-1α, VEGF, RUNX2, Osterix, OCN, and OPN in the four groups at the termination of 4- and 6-week consolidation. The semiquantitative measurements showed that the six markers were highly expressed in Group1, Group2, and Group3 (especially in Group2 and Group3) compared to Control after 4 weeks of consolidation. At the termination of the 6-week consolidation, these indicators were less expressed in Group2 and Group3 compared to Group1 (^#^ *p* < 0.05 vs. Control, *p < 0.05 vs. Group1, **p < 0.01 vs. Group1).

In the immunohistochemical analysis, the expression of HIF-1α, VEGF, RUNX2, Osterix, OCN, and OPN was increased in the three experimental groups after the CDC technique at week 4 when compared with Control (*p* < 0.05). At the same time, these indicators were all more highly expressed in Group2 and Group3 compared to Group1 (*p* < 0.05). However, the aforementioned indicators were less expressed in Group2 and Group3 compared to Group1 at the termination of the 6-week consolidation (*p* < 0.05). (Figure 7).

### The Serum Content of HIF-1α and VEGF

**FIGURE 8:**
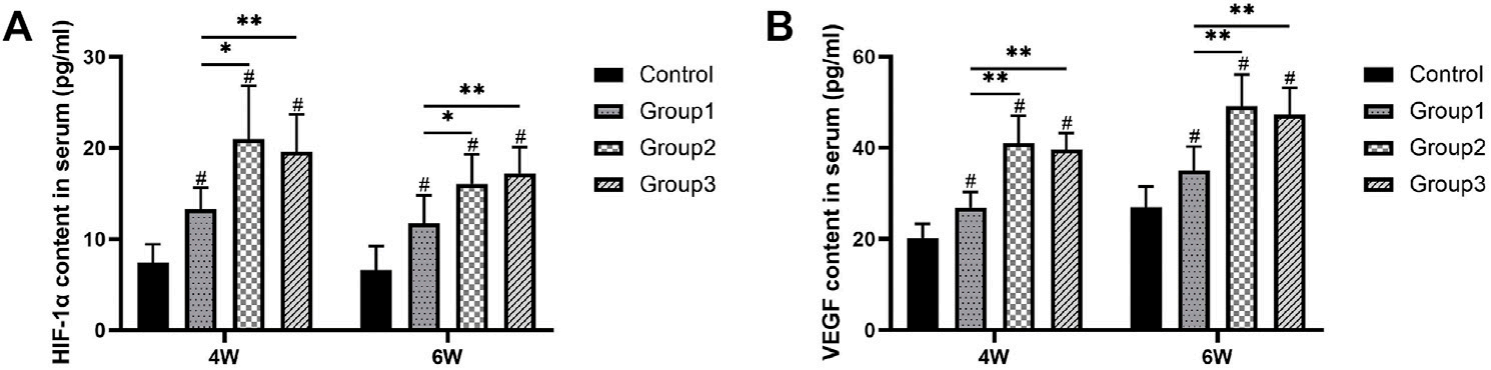
Serum content of HIF-1α and VEGF evaluated by ELISA. The two indicators were higher in Group2 and Group3 than in Group1 after the CDC technique at week 4. Similar results were observed at the termination of the 6-week consolidation (^#^ *p* < 0.05 vs. Control, *p < 0.05 vs. Group1, **p < 0.01 vs. Group1).

After the CDC technique at week 4, the serum content of HIF-1α and VEGF was higher in Group1 (13.26 ± 2.36 and 26.93 ± 3.39 pg/ml, respectively), Group2 (20.98 ± 5.83 and 41.02 ± 6.03 pg/ml, respectively), and Group3 (19.59 ± 4.12 and 39.65 ± 3.58 pg/ml, respectively) compared to Control (7.45 ± 1.99 and 20.21 ± 3.18 pg/ml), respectively (*p* < 0.05). Furthermore, the values of the two indicators in Group2 and Group3 were significantly higher than those in Group1 (*p* < 0.05). Similar results were observed at the termination of the 6-week consolidation; the serum content of the two indicators was also higher in Group1 (11.70 ± 3.10 and 36.70 ± 3.61 pg/ml), Group2 (16.08 ± 3.20 and 49.11 ± 6.99 pg/ml), and Group3 (17.22 ± 2.89 and 47.34 ± 5.83 pg/ml) compared to Control (6.57 ± 2.67 and 26.98 ± 4.52 pg/ml) (*p* < 0.05); HIF-1α and VEGF contents were higher in Group2 and Group3 than in Group1 (*p* < 0.05) (Figure 8).

## Discussion

The CDC (accordion maneuver) dynamization technique had been described by Ilizarov to accelerate bone regenerate during DO (Ilizarov, 1990). The primary principle was that distraction transforms the newly weak callus into the fibrovascular tissue and repeated distraction stimulates the collagen fiber and osteoblastic cell production followed by bone formation via intramembranous ossification (Neidlinger-Wilke et al., 1994). Although several clinical studies reported positive outcomes regarding the application of the accordion maneuver in cases with poor bone formation during DO, the details of when and how this technique is applied were poorly described (Inan et al., 2005; Madhusudhan et al., 2008; Makhdom et al., 2015; Zhang et al., 2017; Liu et al., 2020; Meselhy et al., 2021). In the present study, we investigated the effectiveness and potential mechanism of different amplitudes and rates of CDC technique on bone regeneration in a rat femur DO model. The results demonstrated that the CDC technique has benefits on the acceleration of bone formation during DO, and better outcomes may be achieved by moderately increasing the amplitude and slowing down the rate.

It was reported that moderate, cyclic, predominantly compressive stimulation during or after callus distraction can enhance bone regeneration (Kassis et al., 1996; Claes et al., 2000; Leung et al., 2004). Kassis et al. (1996) confirmed that micromovements applied after the end of elongation were beneficial for bone healing in a rabbit model. Leung et al. (2004) demonstrated the beneficial effect of weight-bearing activity during DO on the enhancement of bone formation. Efforts have been made to understand the effect of the CDC technique on bone regeneration. Interestingly, from a mechanistic approach, it was found that dynamic compression has a greater effect on bone formation than static compression (Saxon et al., 2005). The most likely explanation is that bone cells desensitize promptly from the mechanical stimulation and that resensitization must happen before the cell can transduce any prospective mechanical loads into biochemical signals; therefore, the bone needs a period of “time off” from mechanical loading, which is caused by a CDC technique (Robling et al., 2002). Moreover, it has been demonstrated that various effects on the differentiation of mesenchymal stem cells may be caused by different load types. Compressive load leads to osteogenesis, fibrogenesis, and intramembranous ossification, while distraction load results in chondrogenesis, angiogenesis, and endochondral ossification (Greenwald et al., 2000; Makhdom et al., 2015).

Xu et al. (Xu et al., 2018) have demonstrated that the accordion maneuver during the middle phase of the consolidation period was effective in accelerating new bone formation. Based on their conclusion (Xu et al., 2018), the effects of rate and amplitude of the CDC technique on bone formation during DO were investigated in this present study. The digital radiographic results demonstrated that the bone quality in terms of volume and continuity of the callus was better in Group2 and Group3 than in Group1 at the termination of the 6-week consolidation. Furthermore, bone union with primary recanalization of the medullary cavity was macroscopically observed in Group2 and Group3; a similar phenomenon was shown on the micro-CT examination. The quantitative analysis regarding BMD, BV/TV, and mechanical properties of the distraction zone also confirmed that the moderately larger amplitude (Group2) and slower rate (Group3) of the CDC technique can obviously accelerate bone regeneration. As for the histomorphological assessment, the accelerated bone regeneration was also shown in Group2 and Group3 when compared to Group1. The aforementioned compelling findings foster the CDC technique, especially in larger amplitude and slower rate as an alternative means to optimize bone regeneration in DO.

It is well known that RUNX2, Osx, OCN, and OPN are early or terminal osteogenic markers during osteoblast differentiation and bone regeneration. In this study, at the termination of the 4-week consolidation, the expression of early osteogenic markers (RUNX2 and Osx) was significantly increased after CDC, especially in Group2 and Group3 as well as the terminal markers (OCN and OPN). Interestingly, the four markers were less expressed in Group2 and Group3 compared to Group1 after 6 weeks of consolidation. Bone formation was obviously accelerated in Group2 and Group3 as evidenced from the aforementioned evaluation; we therefore speculated that the more mature regenerate trabeculae in Group2 and Group3 produce less osteogenic relative factors and proteins when compared to Group1.

The finding of this study that moderate larger compressive amplitudes stimulate more bone formation than small amplitudes is in agreement with previously published data (Kassis et al., 1996; Claes et al., 2000; Hente et al., 2004; Leung et al., 2004; Schuelke et al., 2018). Hente et al. (2004) observed that the amount of periosteal callus formation was up to 25 times greater on the compression compared to the distraction side in a sheep tibial model. Schuelke et al. (2018) also concluded that the larger compression amplitude representing moderate compressive interfragmentary movements stimulated significantly more new bone formation than the small compression amplitude. Similar results are observed in other studies in which greater compressive interfragmentary movements led to higher callus density (Claes et al., 1995). These may explain the positive effect of moderate larger amplitude during the accordion maneuver. In addition, the slower rate may lead to a long duration of “time off” from mechanical loading when CDC was applied, resulting in a sufficiently long period for bone cells to become resensitized. This may indirectly explain why the slower rate contributed to the satisfactory outcomes of bone regeneration. Another possible reason might be the capability for the revascularization under different amplitudes and rates of interfragmentary movements as vascularization is a prerequisite for bone formation.

Bone is a highly vascularized and heterogeneous tissue that forms through at least two independent mechanisms: intramembranous and endochondral ossification (Karsenty, 2003). Angiogenesis and osteogenesis are tightly coupled during bone development and regeneration (Danis, 2001; Wan et al., 2008; Schipani et al., 2009; Maes et al., 2012). The vasculature supplies oxygen to developing and regenerating bone and also delivers critical signals to the stroma that stimulate mesenchymal cell specification to promote bone formation, and blood vessel invasion has become a critical step in the replacement of cartilage by bone and the formation of the bone marrow cavity (Schipani et al., 2009). At the center of the distraction gap in DO, cells that exhibit morphological features consistent with osteoblasts are hypoxic and express both HIF-1α and VEGF (Wan et al., 2008). It has been reported that hypoxia is a major driving force for angiogenesis and VEGF expression by stabilizing the HIF protein (Wan et al., 2008; Semenza, 2009).

In the present study, the immunohistochemical analysis manifested that VEGF was highly expressed in osteoblasts and chondrocytes after CDC in Group1, Group2, and Group3 (especially in Group2 and Group3) compared to Control and then decreased with more trabeculae matured in the bone medullary callus. The positive immunoreactivity trend of HIF-1α, which is upstream of VEGF, was also similar to VEGF. Moreover, the serum content of HIF-1α and VEGF also showed higher expression in Group2 and Group3 than in Group1 after different patterns of the CDC technique. Growing evidence has demonstrated that the HIF pathway is a central regulator, which can coordinate the initiation of angiogenesis and couple these events to bone formation. We, therefore, speculated that the cyclic compressive forces by CDC may lead to tissue hypoxia around the center of the distraction gap and trigger the HIF pathway followed by the upregulation of HIF-1α and VEGF expression and the augmentation of osteogenic–angiogenic coupling, resulting in angiogenesis acceleration and osteoblast differentiation.

Interestingly, we also noticed that there was a more advanced remodeling of Group2 compared to Group3 based on the morphological evaluation as well as the quantitative analysis of mechanical properties and regenerate quality in micro-CT. However, the CDC rates and amplitudes are all different between Group2 and Group3, so the final results of the two groups were not statistically comparable from a univariate analysis that was conducted in this study. But in terms of the combined effect under the same stimulation duration, we speculated that the beneficial effect on bone regeneration caused by a larger amplitude due to a faster rate may be stronger than that in a smaller amplitude caused by a slower rate, and further detailed experimental data are needed to confirm this conjecture. In addition, the stimulation duration may be another factor that has an effect on bone regeneration during CDC. In the present study, we slowed down the rate of Group3 to compare the effect of the CDC rate on bone regeneration at the same CDC amplitude, which resulted in a longer stimulation time in Group3 compared to that in Group1. The better bone regeneration in Group3 proved the greater effect of a longer stimulation to some extent. However, univariate analysis is also needed to determine this greater effect, such as increasing the cycles of the CDC technique while controlling the rate and amplitude.

Despite our promising findings, there were several limitations in this study. First of all, only one cycle of compression and distraction was performed in the present study; subsequent investigation is needed to determine whether more cycles of the CDC technique will lead to different outcomes. In addition, the limit of larger amplitude and slower rate needs to be optimized for superior effectiveness. Moreover, histologic and morphologic characteristics of the regenerated bone were used as the main principle for the effectiveness evaluation in this study; future directions may consider the detailed molecular mechanisms that underlie the observed effects. In summary, our results demonstrated that the CDC technique has benefits on the acceleration of bone formation during DO, and better outcomes may be achieved by moderately increasing the amplitude and slowing down the rate.

## Conclusion

The CDC dynamization technique, which is named as the accordion maneuver, has benefits on the improvement of bone formation during DO, and the mechanism may be due to the tissue hypoxia activating the HIF pathway followed by the augmentation of osteogenic–angiogenic coupling. Better outcomes may be achieved by moderately increasing the amplitude and slowing down the rate of the CDC technique. Further elucidation of these mechanisms may contribute to a better understanding of the coupling between angiogenesis and osteogenesis and may suggest promising approaches for the optimization of DO.

## Data Availability

The original contributions presented in the study are included in the article/Supplementary Material, further inquiries can be directed to the corresponding author.
